# Applications of honeybee-derived products in bone tissue engineering

**DOI:** 10.1016/j.bonr.2024.101740

**Published:** 2024-01-19

**Authors:** Shahla Korani, Naeemeh Khalesi, Mitra Korani, Tannaz Jamialahmadi, Amirhossein Sahebkar

**Affiliations:** a Research Center of Oils and Fats, Kermanshah University of Medical Sciences, Kermanshah, Iran; b Biotechnology Department, School of Advanced Technologies in Medicine, Shahid Beheshti University of Medical Science, Tehran, Iran; c Medical Toxicology Research Center, Mashhad University of Medical Sciences, Mashhad, Iran; d Applied Biomedical Research Center, Mashhad University of Medical Sciences, Mashhad, Iran; e Biotechnology Research Center, Pharmaceutical Technology Institute, Mashhad University of Medical Sciences, Mashhad, Iran

**Keywords:** Honey, Propolis, Royal jelly, Bee pollen, Beeswax, Bee venom, Bone engineering, Bone defect

## Abstract

Nowadays, there is an increasing prevalence of bone diseases and defects caused by trauma, cancers, infections, and degenerative and inflammatory conditions. The restoration of bone tissue lost due to trauma, fractures, or surgical removal resulting from locally invasive pathologies requires bone regeneration. As an alternative to conventional treatments, sustainable materials based on natural products, such as honeybee-derived products (honey, propolis, royal jelly, bee pollen, beeswax, and bee venom), could be considered. Honeybee-derived products, particularly honey, have long been recognized for their healing properties. There are a mixture of phytochemicals that offer bone protection through their antimicrobial, antioxidant, and anti-inflammatory properties. This review aims to summarize the current evidence regarding the effects of honeybee-derived products on bone regeneration. In conclusion, honey, propolis, royal jelly, beeswax, and bee venom can potentially serve as natural products for promoting bone health.

## Graphical abstract

**Figure:**
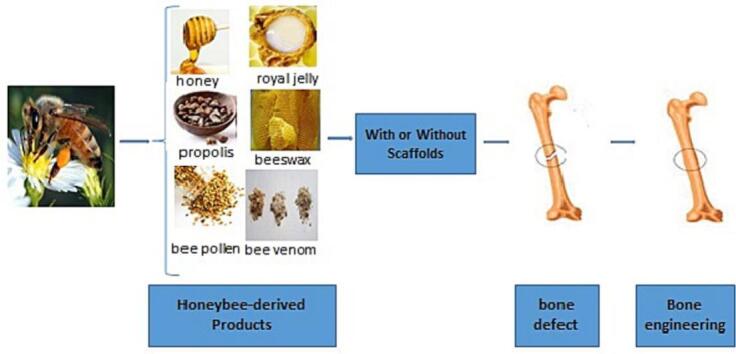

## 1 Introduction

Bone is a vital organ in the body that performs several important functions. An adult human body consists of 206 bones, which not only support weight and facilitate movement but also contribute to blood cell generation, mineral and growth factor storage, protection of the brain and heart, provide surfaces for muscle, ligament, and tendon attachment, and offer mechanical support to various tissues (Clarke, 2008).

Musculoskeletal diseases such as rheumatoid arthritis, osteoarthritis, osteoporosis, low back pain, and limb trauma are increasingly prevalent (Roshanbinfar and Ansari, 2013). With the rise in life expectancy, age-related diseases pose a potential threat to the quality of life in the elderly population (Raquel Maia et al., 2019). Inappropriate bone regeneration is often the cause of many bone fractures (Naghib et al., 2012). Large segmental bone fractures require orthopedic surgeries as they cannot heal naturally. Additionally, spinal fusion operations may result in non-union (Eslami et al., 2018; Sheikh et al., 2019). Bone transplantation is currently the only biomedical method for remodeling bone defects, which can be achieved through autogenic, allogenic, or xenogeneic procedures (Kim et al., 2014a). However, each of these methods is associated with certain complications. Autografting may lead to nerve injury, infections, morbidity, and chronic pain at the surgical sites. Allografting carries the risk of disease transmission, infection, and implant rejection due to immune reactions. Xenografting is also prone to immunogenicity and infection, despite its advantages of easy bone harvesting and rich graft sources compared to the other methods. To address these complications, scientists have been investigating natural or synthetic biomaterials for the appropriate treatment of bone defects (Venkatesan et al., 2015; Goulet et al., 1997; Badylak, 2004; Wu et al., 2014).

Successful bone restoration and functionality require collaboration among different cells and intra- and extracellular signals that induce and guide bone growth (Schroeder and Mosheiff, 2011). While patients often achieve bone defect restoration with significant gaps, tissue engineering aims for ideal regeneration that fully restores the anatomy and missing tissue (Bayat et al., 2015). Proper treatment of the lesions involves regenerating bone with osteoblasts and establishing adequate biological connections with the surrounding bone tissue (Hoffer et al., 2008). Although numerous studies have been conducted, a definitive solution for treating these lesions has not yet been developed.

Tissue engineering utilizes scaffolds, growth factors, and osteoblasts to regenerate lost bone (Bahrami et al., 2017). The most commonly used scaffolds for bone regeneration are allografts, xenografts, and synthetic hydroxyapatite (Bayat et al., 2017). These materials possess a mineral structure similar to bone, providing guidance for regeneration, but they may be insufficient for regenerating critical defect volumes (Mohammadi et al., 2018). Although certain drugs are used in these conditions, they have been shown to cause harmful side effects in patients. Considering the aforementioned points, researchers have recognized the significant potential of organic drugs for application in bone defect treatment.

Honey and bee products have a long history of use in traditional and modern medicine. Honey, bee bread, bee venom, bee pollen, propolis, and royal jelly have been employed in various ways (Martinotti and Ranzato, 2018). This review aims to present the current evidence regarding the effects of Honeybee-derived products on bone regeneration.

## 2 Honey

Honey is an aromatic viscous food with a sweet taste, consisting of approximately 200 ingredients (Machado De-Melo et al., 2018). The primary constituent of honey is sugar, which makes up 76 % of its composition, with monosaccharides being the most abundant. The remaining contents include water (20 %) and other materials. Fructose is more abundant than glucose in the monosaccharide portion, and the disaccharide section is composed of maltose, isomaltose, nigerose, turanose, and maltulose. During storage, the ingredients of honey can undergo degradation when exposed to heat and amino acids, resulting in the formation of products such as 2-acetylfuran, isomaltol, 3,5-dihydroxy-2-methyl-5,6-diidropiran-4-one, and maltol. These changes can affect the taste, odor, and color of honey. The moisture content of honey is also a physical factor that can influence its properties (Escuredo et al., 2014; da Silva et al., 2016).

In addition to sugar, honey contains enzymes, organic acids, vitamins (such as B6, thiamine, niacin, riboflavin, and pantothenic acid), minerals (including calcium, zinc, iron, manganese, magnesium, phosphorus, potassium, sodium, and zinc), pigments, phenolic compounds, organic acids, various volatile compounds, and solid pieces obtained during honey harvesting (da Silva et al., 2016).

Phenolic compounds are the most important antioxidants in honey and contribute to its therapeutic application in the treatment of diseases associated with oxidative stress, such as diabetes mellitus, hypertension, atherosclerosis, cancer, and Alzheimer's disease (Arráez-Román et al., 2006; Moniruzzaman et al., 2014; Erejuwa et al., 2012).

The medicinal use of honey has a long history due to its chemically complex nature. Honey has antioxidant, antimicrobial, and anti-inflammatory properties, making it beneficial for wound care and healing. It influences the immune system, acts as a debridement agent, and stimulates wound regeneration (Al-Ghamdi and Ansari, 2021). Honey with anti-inflammatory activity aids in wound debridement, inhibits scarring, reduces wound exudate, and improves tissue recovery (Khan et al., 2018).

Studies have demonstrated that honey-derived flavonoids and proteins inhibit the production of TNF-α and IL1-β by microglia cells when stimulated with LPS. Similar effects were observed regarding the release of TNF-α by human monocyte cells and the secretion of ROS by macrophages and neutrophils after activation by zymosan (Candiracci et al., 2012; Mesaik et al., 2015).

The antimicrobial properties of honey are associated with peroxidase enzymes, such as glucose oxidase, which produces hydrogen peroxide, as well as non-peroxidase factors, including complex phenols and flavonoids (Almasaudi, 2021). Even in the presence of catalase, which removes hydrogen peroxide, the antimicrobial activity of honey remains, indicating the role of non-peroxidase elements with antimicrobial properties in addition to the contribution of peroxidase factors. These antimicrobial substances are specific to the plant flora that honey bees utilize. The antioxidant components of honey also play a role in eradicating bacterial infections. Furthermore, honey ingredients can contribute to antibody synthesis and the production of cellular components of the immune system, thereby exerting antimicrobial effects (Martinotti and Ranzato, 2018).

In addition to its components, honey's antimicrobial activity is attributed to parameters such as its low pH (ranging from 3.2 to 4.5) and high osmolarity resulting from its high sugar concentration (Singh et al., 2014; Eteraf-Oskouei and Najafi, 2013). The acidic environment of honey is unfavorable for microorganisms, and its high osmolarity can cause osmotic shock, depolarize the cell membranes of microorganisms, increase membrane permeability, and decrease cell size (Masoura et al., 2020). However, studies comparing natural and processed commercial honeys have shown that commercial honeys lack antimicrobial activity, indicating that factors other than sugar content, osmolarity, and pH play a crucial role in this activity (Matzen et al., 2018).

The effectiveness of antimicrobial substances in honey depends on the presence or absence of an outer membrane surrounding bacterial cells. Gram-positive bacteria, for example, are partially protected by their outer membrane against the penetration of antimicrobial factors (Mancuso et al., 2019). In the immune system of invertebrates, antimicrobial peptides such as Defensin-1, produced by honeybee glands, play a role (Cornara et al., 2017; Marrazzo et al., 2019). Defensin-1 acts synergistically with hydrogen peroxide to exert antimicrobial effects. However, when used alone, it may not have a significant effect on certain bacteria such as *Staphylococcus aureus*. An interesting property of Defensin-1 is its ability to induce the production of MMP-9 by keratinocytes, which aids in wound re-epithelialization in vitro and in vivo (Bucekova et al., 2017).

The impressive effects of honey on wound healing have led researchers to explore its potential use in the restoration of hard tissues. Evidence has shown that honey has a positive influence on the proliferation of dental pulp stem cells (Mohamad et al., 2019) and can effectively treat mandibular and calvaria bone defects when used to fill the defects in vivo (Mohamad et al., 2019; Hajizadeh et al., 2018; Asgari and Bahman Derakhshan, 2022). Hajizadeh et al. studied the effects of topical honey on mandibular bone defect healing in Rats. Their histomorphometric evaluation disclosed that honey could accelerate and increase repair of mandibular small defects in rats. (Hajizadeh et al., 2018).

In rats, the design of calvarial bone defects is a standard method to assess the osteogenic effects of numerous materials before animal research or human trials (Spicer et al., 2012). Defects created in maxillofacial bone and the skull, similar to those in the femur, do not require fixation.

Asgari et al. studied the effects of topical use of manuka honey on calvarial bone defect healing in Rats. histological evaluations showed that topical use of Manuka honey enhanced bone regeneration and caused no inflammatory reaction in rats (Asgari and Bahman Derakhshan, 2022). Limitations of these studies were a short follow-up period and a relatively small sample size. In addition, the molecular tests of bone healing was not performed.

However, for large bone defects, biomaterials such as hydroxyapatite that are osteoconductive material with porous structure are typically necessary to aid in the repair process (Najdanović et al., 2015; Najdanović et al., 2016; Živković et al., 2021; Zivkovic et al., 2015; Zivkovic et al., 2016; Vukelić-Nikolić et al., 2018; Najman et al., 2016; Cvetković et al., 2015; Najdanović et al., 2019; Živković et al., 2017) Composition and structure of hydroxyapatite is very similar to that of natural bone and is commonly used in tissue engineering scaffold, mainly for its osteoconductive and osteoinductive properties (Parizi et al., 2013).

In a study on rat radius bone defects, a mixture of honey and hydroxyapatite was more effective in the repair process compared to using either hydroxyapatite or honey alone (Bigham Sadegh et al., 2018). The limitation of this study was the small number of samples; also, in this study molecular tests of bone healing was not carried out.

In a small clinical trial, honey was applied immediately after surgical removal of impacted third molars, resulting in reduced pain, post-surgical complications, and swelling in patients (Elbagoury and Fayed, 1985).

Since honey appears to regulate immune responses, it can be used as an additive to scaffold materials in individuals with bone infections (Hixon et al., 2018). Evidence suggests that Manuka honey reduces inflammation, promotes collagen deposition and fibroblast migration, and acts as a potent promoter of tissue/material integration and regeneration, accelerating tissue healing around the wound (Leong et al., 2012; Ranzato et al., 2012; Sell et al., 2012). Cryogels, which maintain a three-dimensional structure with proper porosity and elasticity, have been considered attractive tools in bone tissue engineering. Recently, the incorporation of honey into cryogels has gained interest as a means to prevent bacterial contamination (Minden-Birkenmaier and Bowlin, 2018). Hixon et al. incorporated different amounts of Manuka honey into gelatin or silk fibroin cryogel mineralized scaffolds. The results showed proper porosity, mechanical properties, cell infiltration, and inhibition of bacterial infection when bone healing took place in the presence of 5 % Manuka honey in silk fibroin cryogel. These characteristics are crucial for bone tissue regeneration (Hixon et al., 2018). The same group also reported that honey had no effect on MG-63 osteosarcoma cell mineralization in vitro (Hixon et al., 2018).

The 45S5 BG scaffold, produced through the foam replication technique (Chen and Boccaccini, 2006), has been extensively studied for its use in bone tissue engineering and as a bone substitute material. Coating scaffolds with polymers is a common method to combine drug delivery carriers with scaffolds in bone tissue engineering. Zein, a corn protein, is a biodegradable and biocompatible polymer known for its film-forming abilities and has been used in tissue engineering and as a drug delivery carrier. A new scaffold has been developed by coating the 45S5 BG scaffold with the biopolymer zein, which is incorporated with manuka honey as an antibacterial agent (Arango-Ospina et al., 2021).

PU has been widely used in tissue engineering due to its flexibility, barrier properties, and oxygen permeability (Khil et al., 2003). PU has also been produced by incorporating olive oil, honey, and propolis and studied for its application in bone tissue engineering (Jaganathan et al., 2018). Additionally, electrospun honey nanofibers incorporated with garlic, edible mushrooms, and mint have been prepared and shown to have beneficial properties for bone and skin repair and regeneration (Shahbazi and Bahrami, 2019).

Studies have shown that the addition of honey and propolis to electrospun membranes enhances their hydrophilic properties (Kim et al., 2014b). It has been observed that electrospun PU/olive oil exhibits hydrophobic behavior, while PU/olive oil/honey/propolis scaffolds demonstrate hydrophilic properties. These synthetic nanocomposites have also shown increased clotting time in coagulation studies. The fabricated scaffolds appear to be safe for red blood cells, as they have a lower hemolytic index percentage compared to the control. HDF cells have shown higher viability rates when in the presence of the developed scaffold compared to intact PU. Overall, the addition of olive oil, honey, and propolis to PU alters the physicochemical and biological nature of the scaffold, making it an acceptable candidate for bone tissue engineering (Jaganathan et al., 2018).

Investigations have indicated that nano-honey scaffolds increase trabeculation and reduce the number of neutrophils when applied in rats with a defect in the parietal bone of their calvarium. Adding the stimulant of regenerating bones and osteoconductive elements in order to evaluate the effects on bone restoration is recommended. Also molecular tests are suggested (Mohammadi et al., 2018). Further experiments have shown that honey has positive effects on the reconstruction of hard and soft tissues in dental sockets, as assessed by the Turnbull, Landry, and Howley indices (Hemalatha, 2015). In a study, the effects of honey, curcumin, ginger, *nigella sativa* and their mixture on bone repair were evaluated by histopathology and biomechanical examinations. Application of honey, curcumin, ginger, and *Nigella sativa* exhibited a beneficial effect on promoting the bone healing in this study. The beneficial effects of honey(oral), the mixture of honey and *Nigella sativa* (topical), and the mixture of honey and curcumin (oral), have also been observed in the restoration of radial bone defects in rats (Mahmoudi-Fard et al., 2022), as well as significant improvement in trabecular bone microstructure in rats with glucocorticoid-induced osteoporosis after 6 to 8 weeks of treatment with 200 mg/kg body weight of honey (Kamaruzzaman et al., 2019; Zaid et al., 2012). In a recent study by Harley et al., it was demonstrated that mineralized collagen scaffolds soaked in 5 % *v*/v manuka honey can increase mesenchymal stem cell osteogenesis. Adding molecular studies in order to assess the effects on bone restoration is suggested. It seems that more studies should be done on parameters such as resorption markers, also bone formation should be assessed, and bone DEXA scan, dynamic histomorphometry, and bone biomechanical tests should be performed, so that we would be able to conclude more complete information. These scaffolds also promote mineral production and inhibit bacterial growth, including the emergence of biofilms (Dewey et al., 2022).

## 3 Propolis

Propolis, often referred to as “bee glue,” is the second most important substance produced by honeybees. It is a resinous material composed of plant components collected by honeybees from various parts of plants, along with honeybee salivary enzymes. Resins and balms, which contain phenolic compounds, make up 50–60 % of propolis, while the remaining ingredients consist of waxes and fatty acids (30–40 %), essential oil (5–10 %), pollen (5 %), and other materials such as amino acids and vitamins (Boisard et al., 2014).

According to published data, more than 300 components have been identified in propolis. These compounds belong to subsets of polyphenols, terpenoids, steroids, sugars, and amino acids (Sun et al., 2015; Graikou et al., 2016).

Flavonoids, aromatic acids, and phenolic esters, such as CAPE, are known as the main ingredients of propolis (Natarajan et al., 1996; Márquez et al., 2004). These compounds belong to the polyphenolic fraction, and the biological activity of propolis depends on them (Bankova et al., 2000). CAPE has been purified and studied both in vitro and in vivo. Data show that CAPE strongly inhibits the transcription factor NF-κB (Natarajan et al., 1996; Márquez et al., 2004). Specifically, CAPE suppresses RANKL-stimulated NF-κB and inhibits the activation of NFAT (Ang et al., 2009), thereby negatively controlling osteoclasts by impeding osteoclastogenesis and inducing apoptosis (Ang et al., 2009).

**Fig. 1:**
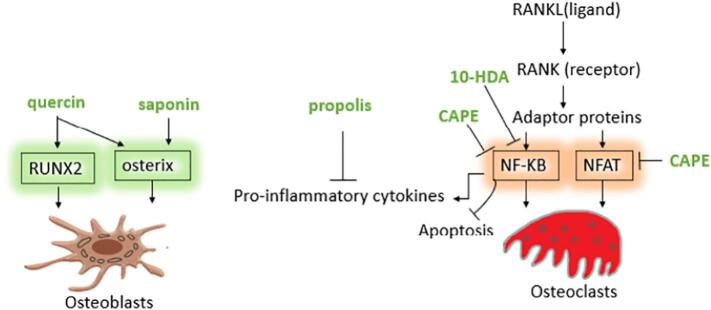
Representative propolis and its components (CAPE, quercin, saponin) and royal jelly component (10HDA) effects on important pathways in osteogenesis. Propolis components inhibit RANKL- induced transcription factors NF-κB and NFAT that are essential in osteoclasts differentiation; and induce transcription factors RUNX2 and osterix that are essential in osteoblast differentiation; propolis induce apoptosis in osteoclasts and regulate inflammation; 10-HDA inhibits RANKL-induced NF-κB.

Bone homeostasis is modulated by cytokines like RANKL, which interacts with the RANK receptor, promoting NF-κB and NFAT signaling in osteoclast cells (Fig. 1) (Xu et al., 2009; Lee and Kim, 2003). In vitro studies have shown that CAPE can inhibit osteoclastogenesis in bone marrow progenitor cells and can stimulate osteoblast differentiation. (Ang et al., 2009). Similar effects have been observed in an ovariectomized mouse model. It would be better that more study was done to understand CAPE signaling pathway in osteoblast differentiation (Wu et al., 2012a). Further data confirm that CAPE interferes with osteoclastogenesis while protecting osteoblasts by affecting the RANKL/OPG pathway and providing protection against osteoporosis in glucocorticoid-treated rats. In this study, histopathological and molecular examinations were performed to demonstrate the effect of CAPE on bone resorption in animals (RANKL/OPG signals and oxidative stress) (Tolba et al., 2017). In a study, the application of 10 % propolis combined with carbonated hydroxyapatite in OFD treatment reduced RANKL protein in rabbits' alveolar bone. Limitations of this study was a short duration of the experiment. It was essential to conduct more research on the impact of the application of 10 % propolis and carbonate hydroxyapatite in OFD with a longer duration of observation (Devitaningtyas et al., 2020).

Interestingly, the effectiveness of CAPE on bone and cartilage regeneration in vivo and its anticancer properties have been revealed in other investigations (Uçan et al., 2013; Kazancioglu et al., 2015; Elmali et al., 2002; Omene et al., 2013; Omene et al., 2012). Additional in vivo studies have shown that CAPE has bone healing properties in a rat calvaria defect model (Tolba et al., 2017) and improves the microstructure of the tibial metaphysis while reducing osteoclast formation in ovariectomized mice with bone loss. (Duan et al., 2014). A study also demonstrates its healing effects on tooth extraction sockets when used systematically (Günay et al., 2014). It is suggested that before designing a clinical trial, studies should be done using different doses of CAPE and a larger number of animals.

Researchers have found that the flavonoids and antioxidant phenols of propolis can remove free radicals, thereby protecting cell membrane lipids and vitamin C from oxidation and degradation. Free radicals disrupt normal metabolism and contribute to cell aging, degradation, and various disease conditions such as heart attack, stroke, arthritis, cancer, diabetes, and Alzheimer's disease (Krol et al., 1990; Scheller et al., 1977; Scheller et al., 1990; Velazquez et al., 2007; Viuda-Martos et al., 2008; Moreira et al., 2008). There is evidence indicating the effectiveness of propolis in the treatment of the common cold (Szmeja et al., 1989) and chronic tonsillitis (Doroshenko, 1983), as well as its application in the cosmetic industry. Phenolic compounds of propolis exert their antimicrobial effects by destroying the microbial membrane, impeding nucleic acid synthesis, and inhibiting biofilm formation (Scatolini et al., 2018).

In an injured tissue, macrophages play a vital role in both regeneration and inflammation through the secretion of regulatory cytokines and inflammatory cytokines/NO (Labonte et al., 2014; Epelman et al., 2014). It has been demonstrated that propolis suppresses the secretion of inflammatory cytokines such as IL-12, IL-6, GM-CSF, IFN-γ, IL-1β, and TNF-α, while increasing modulatory cytokines IL-4, IL-10, and TGF-β (Franchin et al., 2012; Machado et al., 2012; Bueno-Silva et al., 2015). Additionally, propolis exerts a negative effect on neutrophil migration (Franchin et al., 2016). Animal studies have indicated that ethanolic extracts of propolis can decrease acute and chronic inflammatory responses (Silva et al., 2004; Ivanovska et al., 1995; Park and Kahng, 1999; Park et al., 1996; Dimov et al., 1991; Moriyasu et al., 1994). In vivo observations have shown a decreased amount of TNF-α and IL-1β secretion and an increased level of osseointegration when TiO2 nanotubes were loaded with propolis and used as Ti dental implants in rats. These implants increased the expression of collagen fibers, osteogenic differentiation proteins such as BMP-2 and BMP-7 in the rat mandible (Somsanith et al., 2018), Propolis, as a drug-delivery system, blocked peri-implantitis and inhibited early inflammation. The process of chemical bonding between metal and propolis should be elucidated, and the impact of surface pretreatment of Ti on the attachment of propolis should be studied further (Somsanith et al., 2018). The flavonoid components of propolis have been found to inhibit inflammation of dental pulp and stimulate the improvement of dentin. In this study, more investigations are needed, including the molecular investigation of the inhibitory effect of propolis on the dental pulp in rats (Sabir et al., 2005).

Data show that the flavonoids present in propolis induce the expression of FGF-2, VEGF-A, osterix, RUNX2, and ALP (Jacob et al., 2015; Conti et al., 2013). FGF-2 is a growth factor with pleiotropic effects that stimulates fibroblast cells and progenitor osteoblasts. Osteoblasts have been recognized to express FGF-2 during fracture repair, indicating their important role in bone fracture repair, bone remodeling, and osteogenesis. Interesting findings show the wound healing effect of propolis by augmentation of FGF-2 formation (Jacob et al., 2015). Transcription factors RUNX2 and osterix are essential for osteoblast differentiation, and their expression is stimulated by quercetin and saponin, two important ingredients in propolis (Fig. 1) (Komori, 2020; Zhou et al., 2010). These proteins promote bone formation by inhibiting lipopolysaccharide (Wang et al., 2014). Saponin, another compound in propolis, induces osteogenic ALP and RUNX2, promoting mineralization (Ke et al., 2016). In addition, cinnamic acid in propolis augments ALP activity and calcium, promoting bone formation while suppressing NF-κB and TNF-α (Conti et al., 2013). Phenolic compounds of propolis are able to stimulate bone regeneration, and this capacity, in addition to the mentioned points, can be due to their regulatory effects on the accumulation of collagen (type I and III) and their positive effect on the deposition of chondroitin sulfate and hyaluronic acid at the site of tissue injury (Olczyk et al., 2013a; Olczyk et al., 2013b).

Experiments have shown that propolis regulates the expression of bone formation markers such as osteocalcin and osteoprotegrin positively, while modulating the expression of resorption markers negatively (Al-Saeed, 2015). Based on other in vivo experiments, it has been revealed that propolis and its extracts are capable of regenerating bone by influencing osteoblasts in a positive way and osteoclasts in a negative way. Limitations of these studies included the lack of histological assessment in the early healing stage that could have revealed initial tissue reaction to propolis material, and also, the absence of clinical findings on the periodontal response to the grafted materials. Therefore, a clinical trial should be considered in future attempts (Zohery et al., 2018; Zohery et al., 2017). Alcoholic extracts of propolis expedite tissue regeneration (Sutta et al., 1975), enhance the healing process in damaged cartilage (Scheller et al., 1977), and increase ossification in induced bone defects (Stojko et al., 1978). These extracts also assist dental pulp regeneration, decline degeneration and inflammation (Scheller et al., 1978). Systemic application of propolis has been shown to stimulate new bone formation in the pre-maxillae in a rat model. Propolis was suggested as a natural and inexpensive product that supports the immune system, with the potential to enhance bone formation at the expanded suture without causing any side effects. The limitations of this study were the small number of samples and a short follow-up period (Altan et al., 2013). Histological information has revealed that propolis causes lower inflammation and more teeth with dentin bridge formation compared to Calcium Hydroxide when these materials are used for pulp capping in human premolars. Assessments have also shown that direct pulp capping with propolis resulted in a pulpal response comparable to that with MTA (Sabir et al., 2005; Parolia et al., 2010). When propolis was used in infected mature and immature dog's teeth, it stimulated the expression of VEGF and VIII factor and was introduced as an appropriate biomaterial for regenerating teeth with closed apices (Zarei et al., 2017).

Propolis has been found to be applicable for controlling and preventing gingival and periodontal diseases by inhibiting plaque formation and periodontal tissue destruction, making it a potential candidate for bone grafting in periodontal defects. Considering the limitations of experimental animal models, studies have been conducted to assess the role of administered propolis in the alleviation of experimental periodontal inflammation. A limitation of the conducted studies has been the short follow-up duration (Szmeja et al., 1989; Doroshenko, 1983; Hegazi and Abd El Hady, 2002; Pereira et al., 2011; Toker et al., 2008). In chronic inflammation conditions like periodontitis, the alveolar bone may be affected due to inflammation in the periodontal tissues (Newman et al., 2012). In such cases, bone grafting using carbonated hydroxyapatite, a biocompatible and osteoconductive alloplastic material, is performed. Integration of propolis into hydroxyapatite is believed to provide antimicrobial and bone regenerating effects. Topical administration of 10 % propolis gel has shown to enhance the improvement of gingivitis and chronic periodontitis in animal models, reducing the number of PMN cells and increasing osteoblast formation and angiogenesis during the healing process (Phd et al., 2017). Other in vivo studies have also indicated propolis to prevent bone loss resulting from periodontitis (Toker et al., 2008; Yuanita et al., 2018), orthodontic tooth movement (Kresnoadi et al., 2020a; Kresnoadi et al., 2020b; Wiwekowati et al., 2020), and implantation (Somsanith et al., 2018). Propolis has also demonstrated beneficial effects on delayed tooth replantation (Gulinelli et al., 2008). In these studies, molecular tests were used as confirmation of immunohistochemical and histological evaluations.

Researchers have investigated the combination of propolis mixture and BBG for inducing alveolar bone formation in tooth extraction sockets. BBG is used as a xenograft to stimulate cell division in fibroblasts, osteoblasts, and endothelial cells (Kresnoadi et al., 2020a). Further studies have shown that this combination stimulates the expression of TGF-β, IL-1β, RUNX2, and ALP, and promotes the regeneration of alveolar bone in tooth extraction sockets (Kresnoadi, 2020). The expression of proteins such as SRT box transcription factors (SOX2 and SOX9) and woven bone is also elevated, indicating increased growth of osteoblasts. SOX2 is necessary for the sustained survival of osteoblasts, while protein SOX9 is essential for osteogenesis. These findings suggest that the combination of BBG and propolis may be beneficial for alveolar bone regeneration. This research needs longer time to observe the bone healing completely (Kresnoadi et al., 2022a).

Propolis extracts have also been used in orthopedic applications to dampen inflammatory responses and promote the reconstruction of osseous tissue in conditions such as purulent inflammation (Scheller et al., 1977). In one study, a fine layer of commercial propolis was applied to an allograft, which was then implanted in the femur of dogs. Commonly healing autograft host interfaces occurs in a shorter period than allografts. This system enhanced and accelerated osteointegration at the proximal and distal junctions before the allograft failed. Complete consolidation occurred at 10 to 12 weeks without new bone remodeling or allograft resorption (Boudra, 2014). Similar results were obtained in another study that used alveolex (containing 10 % propolis) in combination with rhBMP-2 in rats with calvaria bone defects. The study showed an improved effect on bone repair when alveolex was combined with rhBMP-2. The alveolex components may have strengthened the rhBMP-2 maintaining on bone defects. In these conditions, Alveolex could be considered as a tool for sustained delivery of rhBMP-2 on the defect area. (Pereira et al., 2012). Additional findings have indicated that propolis can decrease osteoclastogenesis when administered in combination with carbonated hydroxyapatite (Devitaningtyas et al., 2020).

Radiological and histological assessments, as well as bone density measurements in in vivo studies, have shown the reparative effect of orally administered propolis and intraperitoneally administered CAPE in rat models of femoral and cranial defects, respectively. Recent investigational evidence suggests potential favorable impacts of propolis in several clinical conditions, including orthopedic practice (Uçan et al., 2013; Guney et al., 2011; Darmadi, 2016). These findings indicate that propolis has potential therapeutic efficacy in osteoporotic bone diseases. Studies have confirmed the healing properties of propolis in diabetic rats (Al-Hariri et al., 2011; NYM, 2015), valproate-treated epileptic rats (Elwakkad et al., 2008), and ovariectomized rats as a menopause model (Juwita et al., 2021). In the case of epileptic rats, propolis administration advanced immunological bone formation markers, such as osteocalcin and bone ALP, while reducing bone resorption markers. This was accompanied by an increase in OPG and a decrease in RANKL expression, resulting in dampened osteoclastogenesis. Further studies are suggested to confirm the direct regulatory impact of propolis on mRNA expression level and production of osteoprotegerin, and to recognize the active element responsible for this impact. (Elwakkad et al., 2008). In diabetic rats, propolis at doses of 300 and 600 mg/kg inhibited oxidative stress and prevented diabetic osteopathy (Al-Hariri et al., 2011). Oral administration of propolis at a dose of 360 mg/kg increased the ratio of osteoblasts to osteoclasts in the femoral metaphysis of ovariectomized rat models of osteoporosis, although it did not affect the thickness of the femoral metaphyseal cortical bone. This study assessed the histological changes and the cortical bone thicknesses, but more analysis such as in vitro assays, scanning electron microscopy (SEM) analysis, in vivo scans, power examinations, and also more samples are needed to evaluate bone microarchitecture, osteoclastogenesis, and morphology. If these limitations are resolved in the future, the mechanisms of action of propolis in preventing osteoporosis in menopausal women could be completely revealed (Juwita et al., 2021).

Propolis has also been used in combination with implantation in animal bone defect models. For example, in rats with critical radial bone injuries, percutaneous injection of watery propolis extract diluted with chitosan or DBM along with the administration of propolis exhibited increased bone remodeling and inhibited the onset of severe inflammatory responses. The lack of in vitro assessment in the primary bone healing phase that could have revealed early tissue reaction to propolis was the limitation of the mentioned study.(Meimandi-Parizi et al., 2018a). The efficacy of propolis in healing femoral defects has also been demonstrated when implants loaded with propolis were used in conjunction with propolis treatment in rats (Guney et al., 2011). Coating implants with propolis has been shown to improve bone formation (Devitaningtyas et al., 2020; Boudra, 2014; Pereira et al., 2012; Al-Molla et al., 2014). Furthermore, new electrospun nanocomposite scaffolds consisting of polyurethane loaded with ghee and propolis have been developed, exhibiting suitable physicochemical properties, blood compatibility, and non-toxicity, making them potential candidates for bone tissue engineering (Mani and Jaganathan, 2020).

## 4 Royal jelly

Royal jelly is secreted by the hypopharyngeal glands of worker bees. The queen bee and larvae are fed this white gelatinous material. Water comprises the main portion of royal jelly (50 %–70 %), while proteins (9 %–18 %), carbohydrates (7 %–18 %), lipids and fatty acids (3 %–8 %), mineral salts (1.5 %), and vitamins (in very small amounts) make up the remaining components (Hattori et al., 2007).

Royal jelly exhibits antimicrobial, antioxidant, anticancer, and anti-inflammatory properties (Hidaka et al., 2006; Kafadar et al., 2012). In vitro and in vivo studies have indicated that royal jelly can mimic estrogen, regulate estrogen receptor activities, and influence osteoblasts and bone metabolism. These activities could potentially improve osteoporosis in ovariectomized rat models and be considered as a treatment for postmenopausal syndrome (Hidaka et al., 2006; Mishima et al., 2005; Moutsatsou et al., 2010; Shimizu et al., 2018). Despite its ability to increase cell proliferation and differentiation, royal jelly also demonstrates antitumor activity. It can induce vascular expansion and improve blood flow. All of these properties make it a suitable candidate for the treatment of age-related diseases (Münstedt et al., 2009; Hassanien, 2012; Sabatini et al., 2009).

Apisin, a major royal jelly glycoprotein, has been investigated and shown to induce the proliferation of neonatal dermal fibroblasts and collagen production. It also stimulates the differentiation of MC3T3-E1 in vitro. These findings suggest that the proliferative and wound healing effects of royal jelly may be associated with the presence of apisin (Siavash et al., 2015). It has been found that royal jelly's activity on estrogen receptors enhances the proliferation of MC3T3-E1 (Kaku et al., 2014). In addition to the aforementioned mitogenic properties of royal jelly, it also induces the production of extracellular bone matrix (Narita et al., 2006; Chen et al., 2016; Musa et al., 2014).

Researchers have demonstrated rapid expansion and regeneration of maxillary bone in rats and believe that the antioxidative action of royal jelly (Özan et al., 2015), along with its ability to induce TGF-β, may be the mechanisms through which this compound exerts its effects on bone tissue (Koya-Miyata et al., 2002; Koya-Miyata et al., 2004). Assessments have revealed that royal jelly can inhibit the decline in bone mineral density and strength resulting from hormone deficiency in ovariectomized rat models (Hattori and Omi, 2021), and it can even reverse osteoporosis induced by glucocorticoids. These studies need to include molecular assays in the future. The components of royal jelly protein have not been assessed in detail. Thus, it is unclear which content of royal jelly protein impacted the bone (MFA, 2022). Reports have shown that partial suppression of bone loss in ovariectomized animals using royal jelly is achieved through the inhibition of osteoclastogenesis. Further experiments have indicated that strong prevention of RANKL-induced osteoclastogenesis can be achieved in the presence of 10HDA, a unique medium-chain fatty acid found in royal jelly. This compound can inhibit bone loss by blocking NF-ƘB signaling (Fig. 1) (Tsuchiya et al., 2020).

Royal jelly induces the expression of osteopontin (a highly phosphorylated sialoprotein), osteocalcin, and ostrix transcripts, and enhances mineralized nodule formation in mouse periodontal ligament cells in vitro (Yanagita et al., 2011). In vitro studies have also shown an upregulation of procollagen I α1 transcription, an increase in calcium uptake, and an enhancement of bone calcium content in the presence of royal jelly (Hidaka et al., 2006). Interestingly, royal jelly has been observed to alleviate lower back pain when used at a dose of 800 mg/day in healthy menopausal women (Asama et al., 2018).

Finally, studies have indicated that a combination of royal jelly and hydroxyapatite or chitosan scaffolds can improve bone reconstruction and healing in animal models (Bigham-Sadegh et al., 2020; Meimandi-Parizi et al., 2018b). This research lacked in vitro test that could be used to reveal the possible mechanisms of action or bioactive ingredients responsible for bone healing.

## 5 Other Honeybee-derived products

### 5.1 Bee pollen

Bee pollen is a mixture of flower pollen, honeybee salivary enzymes, and nectar and is consumed as a food supplement due to its nutritional composition, including proteins, sugars, lipids, essential amino acids, fatty acids, minerals, vitamins, phytosterols, and flavonoids (Sabatini et al., 2009; Siavash et al., 2015; Kaku et al., 2014). Bee pollen contains compounds with antioxidant, anticancer, and immune modulatory effects (Abdelnour et al., 2019; Mărgăoan et al., 2019). It also exhibits antimicrobial properties (Didaras et al., 2020). Researchers have assessed the impact of bee pollen on bone structure and repair.

Some studies have shown that administering 5 and 10 mg/100 g body weight of bee pollen in rats increased the level of calcium in femoral bone. Aqueous extract of bee pollen has also been shown to have an anabolic influence on femoral diaphyseal and metaphyseal bone composition in in vitro and in vivo studies. However, other investigations have reported no effect on calcium concentration and bone mineralization when bee pollen was used as a food supplement in different dosages. These studies showed that the effect of diet supplementation with bee pollen on tibial bones in birds would cause lower calcium (Kleczek et al., 2012; Oliveira et al., 2013). In some cases, supplementation of food with bee pollen has been reported to reduce bone mineralization, length, weight, and thickness in quails and female rats. (Sabatini et al., 2009; Özan et al., 2015). Another study reported negative effects on various bone parameters, such as weight, cortical thickness, calcium concentration, ALP reaction, and the volume of primary and secondary osteons, when bee pollen was used at a level of 0.75 % in rats, while no efficacy was observed at a level of 0.5 %. In this research, a small number of animals were studied, and furthermore, only male rats were used in the experiments. Therefore, it is recommended to increase the sample size and include animals of both sexes in future studies. Additionally, conducting further experiments with different doses of bee pollen and extending the supplementation period would provide valuable insights and enhance our understanding of its effects (Martiniakova et al., 2021).

### 5.2 Bee venom

Bee venom is an odorless substance produced by a bee's abdominal gland and is used as a defense mechanism. It is commercially referred to as apitoxin and contains several bioactive compounds. The main ingredient of bee venom is the peptide melittin, which makes up 50 % of the dry toxin. Other peptides (apamin, adolapin, and mast cell degranulating peptide), enzymes (phospholipase A2 and hyaluronidase), and non-peptide elements (histamine, dopamine, and norepinephrine) make up the remaining part (Wehbe et al., 2019; Raghuraman and Chattopadhyay, 2007; Moreno and Giralt, 2015; Gajski and Garaj-Vrhovac, 2013).

Bee venom has anti-arthritic, anti-tumor, and pain-reducing properties (Raghuraman and Chattopadhyay, 2007; Gajski and Garaj-Vrhovac, 2013; Im et al., 2016) and has been used in medicine for treating patients through in situ injection of lyophilized toxin or direct application of honey bee (Ali, 2012; Anon, 1995). These techniques have shown therapeutic potential in patients with autoimmune diseases (e.g., rheumatoid arthritis, psoriasis), neurological complications, chronic inflammation, pain, skin problems, and infectious diseases (Anon, 1995; Zhang et al., 2018).

Importantly, bee venom can regulate metabolism in bone tissue, and investigators have discovered several mechanisms for this ability. The first mechanism involves dampening the RANK-RANKL signaling pathway and protecting against the effects of IL-1β on osteoclastogenesis (Badr et al., 2016). The second mechanism is exerted through the production of IL-1α and IL-6 as a result of mast cell degradation and the release of histamine and heparin. Mast cells are affected by the hyaluronidase enzyme and phospholipase A in bee venom (Al Subaie et al., 2016; Pak, 2017; Yang et al., 2014). Experiments have indicated that proinflammatory cytokines such as TNF-α and IL-1 play important roles in bone regeneration (Oryan et al., 2014; Rutkowski, 2014; Schmidt-Bleek et al., 2014). The third mechanism is related to the antioxidative properties of bee venom, which can accelerate bone repair processes by reducing reactive oxygen species, nitric oxide, and anti-inflammatory cytokines (Badr et al., 2016). Finally, the next mechanism involves the ability of bee venom to stimulate the production of growth factors such as TGF-β and vascular endothelial growth factor at the site of repair, resulting in cell proliferation and differentiation (Dang et al., 2011; Manivannan et al., 2021; Aron et al., 2012; Hixon et al., 2019; Kresnoadi et al., 2022b).

### 5.3 Bee wax

Bee wax is composed of straight-chain esters and monohydric alcohols that are esterified with straight-chain acids. Bee wax has been Generally Recognized as Safe (GRAS) by the FDA and is considered a nontoxic and nonirritant substance. It is used topically and orally in pharmacological and food products (Dinker et al., 2018).

Bee wax-chitosan biocomposite has shown promising parameters for application in tissue and bone repair (Zhang et al., 2014). Recent studies have evaluated new biocomposites using bee wax-chitosan and zinc-hydroxyapatite nanoparticles (Manivannan et al., 2021).

## 6 Conclusions and discussion

In vivo studies have demonstrated that honeybee products improve bone healing in various animal models and prevent bone loss due to osteoporosis and periodontitis (Table 1). Honeybee products exert their effects through their high antioxidant, anti-inflammatory, and antimicrobial properties when used independently or in combination with different biomaterials as scaffolds. The use of honey, propolis, royal jelly, beeswax, and bee venom can enhance bone regeneration, and their integration with composites such as hydroxyapatite, cryogel, bioactive glass, or nanocomposite polyurethane, as well as their application along with biomaterials like chitosan, DBM, BBG, has improved the properties of these materials for bone engineering.

**Table 1:** Summary of in vivo studies examining honey bee- derived products effects on different bone defect models.

Study	Animal model/size and location of defect	Material/rout	Outcome^⁎^
(Hajizadeh et al., 2018)	Rat/2*2 mm in mandibular angle	Defect was filled with honey	Defect was filled with new bone (new bone formation (%):64.3 & 52.3 in treated vs control after 4 W)
(Asgari and Bahman Derakhshan, 2022)	Rat/Critical sized calvaria defect (8 mm in diameter in the parietal bone at the sagittal suture)	Defect was filled with honey	The regeneration enhanced (new bone formation (%): 30.4 & 13.6 in treated vs control after 8 W)
(Mahmoudi-Fard et al., 2022)	Rat/Radial bone defect (3 mm bone piece was harvested by approaching the midshaft of radius)	Honey or honey -curcumin mixture /orally Honey - *nigella sativa* mixture/ topical in site of suture	Histological & biological result improved
(Kamaruzzaman et al., 2019)	Rat/Glucocorticoid induced osteoporosis	Honey/orally	Oxidative stress & osteoclasts reduced, osteoblasts increased, bone structure maintained
(Bigham Sadegh et al., 2018)	Rat/Radial bone defect (4 mm)	Hydroxyapatite-honey combination/graft	Effective repairing of bone defect (radiographical scores: 4–8-8 & 2–3-5 in treated vs untreated control after 14,28,42,58 days)
(Mohammadi et al., 2018)	Rat/Calvaria defect (depth 5 mm and length 6 mm in parietal bone)	Nano-honey scaffold/graft	More bone trabeculation
(NYM, 2015) (Al-Hariri et al., 2011)	Rat/diabetes induced osteoporosis	Propolis/orally	Bone mineralization improved
(Elwakkad et al., 2008)	Rat/valproate induced osteoporosis in epileptic animal	Propolis/orally	Bone formation markers & OPG increased; bone resorption markers, TNFα,RANKL decreased
(Juwita et al., 2021)	Rat/ ovariectomy induced osteoporosis	Propolis/orally	Ratio of osteoblasts to osteoclasts in the femoral metaphysis increased
(Duan et al., 2014)		CAPE/intraperitoneal	Bone mineral density & microarchitecture improved; osteoclasts decreased in tibial metaphysis
(Wu et al., 2012a)	Mice/ ovariectomy induced osteoporosis	CAPE/intraperitoneal	Osteoclastogenesis and bone loss reduced
(Tolba et al., 2017)	Rat/glucocorticoid induced osteoporosis	CAPE/intraperitoneal	Osteoclastogenesis and bone loss reduced
(Uçan et al., 2013)	Rat/critical cranial defect (7 mm in diameter into the dorsal portion of the parietal bone on each side of the midsagittal suture)	CAPE/ intraperitoneal	Bone healing improved (scores for amounts of fibrous + cartilage + immature bone tissue: 5.93 & 5.36 in treated vs control after 4 w)
(Darmadi, 2016)	Rat/femoral bone fracture	Propolis/orally	Osteoblast & chondrocyte increased; osteoclasts decreased
(Guney et al., 2011)			Fracture healing (bone mineral density (g/cm^2^): 0.18 & 0.14 and 0.19 & 0.15 in treated vs control after 3 & 6w; radiological scores: 3&1.5 and 4&2 in treated vs control after 3 &6w respectively; histological scores: 8 & 6 in treated vs control after 6w); BMD increased; super oxide dismutase, glutation & myeloperoxidase decreased in treated group
(Altan et al., 2013)	Rat/maxillary expansion	Propolis/orogastric tubes	Osteoblasts & bone formation increased; 3 rats out of 7 rats gained max score for bone formation in treated vs 0 out of 7 in control
(Günay et al., 2014)	Rat/tooth extraction socket	CAPE/intraperitoneal	Tooth socket healing improved
(Kresnoadi et al., 2020a) (Kresnoadi et al., 2020b)		BBG-propolis/applied in sockets	FGF2,VEGF,HSP70,osteoblast increased; osteoclasts decreased
(Yuanita et al., 2018)	Rat/post-pulp chronic apical periodontitis infection	Propolis extract/ applied in exposed pulp and cavity filled with glass ionomer cement	OPG increased and osteoclasts decreased
(Sabir et al., 2005)	Rat/ exposed dental pulp		Delayed inflammation and stimulated reparative dentin
(Devitaningtyas et al., 2020)	Rabbit/induced periodontitis	10 % propolis ‑carbonated hydroxyapatite combination/open flap debridement in alveolar bone	RANKL expression decreased
(Toker et al., 2008)	Rat/induced periodontitis	Propolis/systemic	Periodontitis related bone loss reduced
(Somsanith et al., 2018)	Rat/mandibular model	Propolis loaded TiO2 nano tube/implant	Osseointegration increased, IL1β & TNFα decreased, Bone mineral density (g/cm^2^) & bone formation (mm ^3^) increased in all time points, e.g., 11.2 & 9.7 and 12.2 & 9.6 in treated vs control respectively after 4 w
(Zohery et al., 2017)	Dog/grade II furcation in mandibular premolar(3*5 mm in mandibular P3&P4)	Propolis/ applied in defect and covered with collagen membrane	Higher bone regenerative effect (%) compared to nano bone (86.79 & 82.48 in treated vs control after 1 m)
(Zohery et al., 2018)			Favorable periodontal regenerative effect (%) compared to nano bone (75.1 % & 89.3 and 74.8 & 80.6 in treated vs control after 1 & 3 m respectively)
(Kresnoadi, 2020)	Cavia cobaya/tooth extraction socket	Propolis extract-BBG^a^ combination/graft	TGFβ increased, IL1β decreased, Alveolar Bone Regeneration
(Kresnoadi et al., 2020a)			FGF2, VEGF, osteoblasts Increased
(Kresnoadi et al., 2022b)			RUNX2, ALP increased
(Kresnoadi et al., 2022a)			SOX2, SOX9, woven bone increased
(Meimandi-Parizi et al., 2018a)	Rat/radial bone defect (critical sized:5 mm bone piece was harvested by approaching the midshaft of radius)	“Aqueous propolis extract/percutaneous in site of defect” *Along with* “Demineralized bone matrix/graft”	Bone healing improved (radiographically bone healing scores: [2.2 & 4 & 4.9] vs [1.5 & 2.9 & 3] and [1&1.8&3.5] in treated vs no treated and DBM after 28, 42 and 56 days respectively) and was comparable to autografting based on Histomorphometrical findings and biomechanical test
(Pereira et al., 2012)	Rat/ calvaria bone defect (5 mm in the left posterior region [parietal bone])	Alveolex (10 % propolis)- rhBMP-2 combination/ graft	Bone repair (%) increased significantly compared to control (coagulum): 10.20 vs 17.2, but not significantly compared to rhBM2 group
(Boudra, 2014)	Dog/femoral segmental bone loss	Orthotopic Allograft (1 cm)-propolis combination/implant	Osteointegration accelerated and increased, full consolidation before remodeling started and allograft resorbed
(Al-Molla et al., 2014)	Rabbit/tibial defect	Propolis-CP Ti^b^ /implant	Early bone formation, mineralization, and maturation. Osseointegration increased
(Kleczek et al., 2012)	Chicken/healthy	Pollen-propolis combination/orally	Higher values of geometric parameters in tibia
(Narita et al., 2006)	Mice/healthy	Royal jelly/orally	Type I collagen & bone formation were induced
(Hidaka et al., 2006) (Kafadar et al., 2012) (Shimizu et al., 2018) (Mishima et al., 2005) (Tsuchiya et al., 2020) (Hattori and Omi, 2021)	Rat/ovariectomy induced osteoporosis	Royal jelly/orally	Sex hormone related bone abnormality reduced
(MFA, 2022)	Rat/glucocorticoid induced osteoporosis	Royal jelly/orally	Osteoporosis was reversed
(Özan et al., 2015)	Rat/maxillary expansion	Royal jelly/orally	New bone formation, osteoclasts, osteoblasts, and capillaries increased
(Meimandi-Parizi et al., 2018b)	Rat/ critical sized radial bone defect (full thickness bone defects with a length of 5 mm)	“Royal jelly/percutaneous in site of defect” *Along with* “chitosan/graft” *Note: in this study effect of bee venom along with chitosan was also assessed and results (except to biomechanical outcomes were comparable to royal jelly)*	Density of osseous tissue (%) increased (after 56 days): 51.6 vs 24.1 and 34.6 in test vs chitosan and autograft groups respectively. Radiographical scores (after 56 days) increased: 6.7 vs 3.7 and 3 in test compared to untreated and chitosan groups respectively. The biomechanical scores increased in test vs autograft group.
(Bigham-Sadegh et al., 2020)	Rabbit/ mid radius bone defect	Hydroxyapatite-royal jelly combination/graft	Bone formation improved (radiographical scores: 6 & 8 & 9 vs 2 & 4 & 3 in treated vs untreated after 2, 4, 6 w respectively)

a Bovine bone grafts.

b Commercially purred titanium.

Some studies have reported no effect or even poorer results (in all time points after surgery) when propolis (10 %) (Pereira et al., 2012) or honey (Bigham Sadegh et al., 2018) are used alone compared to untreated control groups. However, these same studies have indicated that in certain time points (e.g., the second week after surgery), the results of honey can be superior to autograft when used in combination with autograft. Furthermore, analysis has shown that the Propolis (10 %) group is better than the untreated group when combined with RhBM2 (Bigham Sadegh et al., 2018; Pereira et al., 2012). These findings underscore the importance of designing different control groups and assessing the effects at different time points in studies.

In some studies, the effects of honeybee products on bone healing have been compared to other products. For instance, honey has demonstrated poorer results compared to hydroxyapatite or autograft in all time points after surgery (Bigham Sadegh et al., 2018). Similarly, propolis (10 %) and RhBM2 showed no significant differences when used independently (Pereira et al., 2012). On the other hand, analyses have shown that chitosan exhibits better radiographic and histomorphometric outcomes when used in combination with royal jelly or bee venom. In fact, the histomorphometric and biomechanical results of chitosan were even superior to the autograft group when applied in combination with royal jelly (Meimandi-Parizi et al., 2018b). Additionally, comparisons have shown superior outcomes of propolis compared to NanoBone and Nanohydroxyapatite (Zohery et al., 2018; Zohery et al., 2017).

In this review, our aim was to investigate the effects of honeybee products on bone healing and regeneration. With the exception of bee pollen, where inconsistent evidence exists with some studies showing no effects or negative effects on various bone parameters, we did not come across data reporting negative effects of these products in studies assessing their impact on bone. However, there are studies reporting cytotoxic effects of honey on different cell lines (fibroblast, pulmonary microvascular endothelial, and macrophage) when applied at concentrations higher than 5 % *v*/v in vitro (Sell et al., 2012). Additionally, honey has been found to have proinflammatory and ototoxic effects on chinchilla ears at a concentration of 50 % v/v (Aron et al., 2012). These findings should be taken into account, and further evaluations regarding the therapeutic application of honey in bone healing and regeneration in vivo should be conducted.

The limitations of most of the studies reviewed in previous sections include the use of unisex animals, small sample sizes, and short evaluation periods. Therefore, it is recommended to use a larger number of animals of both sexes. Additionally, performing in vitro experiments alongside in vivo experiments and evaluating molecular tests of bone healing would be beneficial. Finally, more comprehensive analyses should be conducted on factors such as resorption markers, bone formation, dynamic histomorphometric parameters, and bone biomechanical strength to draw more robust conclusions.

Due to the limited availability of clinical studies, the applicability of these findings to humans remains uncertain. The lack of human data significantly hampers the assessment of the therapeutic potential of honeybee products for bone regeneration. Rigorously designed randomized controlled trials are necessary to validate the effectiveness of honeybee product supplementation on bone health in humans. There are several challenges associated with the clinical application of honey. For instance, when honey is used in wound treatment, direct application may introduce potential toxicity concerns (Minden-Birkenmaier and Bowlin, 2018). Therefore, caution should be exercised to avoid necrosis in adjacent tissues when using honey in bone applications (Minden-Birkenmaier and Bowlin, 2018). As discussed in previous sections, incorporating honey into biomaterials such as electrospun fibers, cryogels, and hydrogels is a crucial aspect of its application in tissue engineering. This approach serves as a viable alternative for delivering honey. However, achieving an optimal concentration of honey in biomaterials poses a challenge in tissue engineering. Innovative methods need to be developed to regulate and prolong the release of honey over time, thereby minimizing cytotoxicity and maintaining stable and effective outcomes over an extended duration. It should be noted that the addition of honey in electrospinning processes can impact the morphology of the fibers, and there is inconsistency in the results. Water-based solutions appear to prevent fiber drying during the electrospinning process. Another important consideration is the type of honey used, as different types of honey can exhibit varying levels of activity. Therefore, clear information regarding the specific type of honey used should be provided in clinical studies (Minden-Birkenmaier and Bowlin, 2018; Hixon et al., 2019). All the aforementioned challenges associated with the use of honey in clinical studies should be carefully considered in the context of bone tissue engineering. However, the utilization of scaffolds offers a potential method for effective delivery of honey in research investigations (Hixon et al., 2019).

Finally, combining honeybee products may have a greater impact on the bone healing process. However, there is a limited number of studies that have investigated the activity of these products in combination, as most studies have focused on the properties of each product individually. It is possible that these products have enhanced properties when used together, and further research should explore this direction. In conclusion, honey, propolis, royal jelly, beeswax, and bee venom may be potential bio products for bone regeneration yet more in vivo and clinical studies are needed to support existing evidence.

## Funding

None.

## CRediT authorship contribution statement

**Shahla Korani:** Writing – original draft. **Naeemeh Khalesi:** Writing – review & editing. **Mitra Korani:** Writing – review & editing. **Tannaz Jamialahmadi:** Writing – review & editing. **Amirhossein Sahebkar:** Writing – review & editing, Conceptualization.

## Declaration of competing interest

None.

## Data Availability

No data was used for the research described in the article.
